# Immune‐Enhancing Effects of IBP35, a Combination of *Lactiplantibacillus plantarum* and *Ligilactobacillus salivarius*, in Macrophages and Cyclophosphamide‐Induced Immunosuppressed Mice

**DOI:** 10.1002/fsn3.71831

**Published:** 2026-04-29

**Authors:** Han Bin Lee, Jinho Lee, Kyuho Jeong, Young Hoon Jung, Jin Seok Moon

**Affiliations:** ^1^ Ildong Bioscience Pyeongtaek‐si Republic of Korea; ^2^ Ildong Pharmaceutical Co., Ltd. Seoul Republic of Korea; ^3^ School of Food Science and Biotechnology, Food and Bio‐Industry Institute Kyungpook National University Daegu Republic of Korea

**Keywords:** cyclophosphamide, IBP35, immunomodulatory activity, *Lactiplantibacillus plantarum*, *Ligilactobacillus salivarius*, macrophages

## Abstract

Rational probiotic combinations should be supported by evidence of enhanced effects of the combined formulation and validated by in vivo efficacy. In this study, we investigated the immunostimulatory potential of IBP35, a 1:1 combination of *Lactiplantibacillus plantarum* IDCC 3501 and *Ligilactobacillus salivarius* IDCC 3551, using in vitro assays and in vivo experiments. In RAW 264.7 macrophages, IBP35 significantly increased nitric oxide production in a dose‐dependent manner, achieving levels 25%–43% higher than those induced by either strain alone (*p* < 0.01–0.001). IBP35 also increased TNF‐α and IL‐6 secretion by 2.0–2.5‐fold compared with individual strains (*p* < 0.001), indicating enhanced macrophage activation. IBP35 was evaluated using a cyclophosphamide (CPA)‐induced immunosuppressed mouse model. CPA administration markedly reduced splenic natural killer (NK) cell activity (46.43% ± 10.64% vs. 62.47% ± 9.53% in normal controls, *p* < 0.05), whereas high‐dose IBP35 significantly increased NK cell activity compared with the CPA group, reaching a level comparable to the normal control group (61.3% ± 7.93%, *p* < 0.05 vs. CPA). In CPA‐treated mice, IBP35 significantly enhanced ConA‐stimulated cytokine production, increasing IFN‐γ, IL‐2, and TNF‐α levels by 2.0–3.0‐fold relative to the CPA group (*p* < 0.01–0.001). Macrophage lysosomal enzyme activity was also significantly elevated (124% of control, *p* < 0.001). Histological analysis revealed that IBP35 attenuated CPA‐induced splenic atrophy and lymphoid depletion. In healthy mice, IBP35 modulated immune responses without inducing excessive inflammation. Collectively, these results indicate that IBP35 modulates both innate and adaptive immune readouts and supports immune function under immunosuppressed conditions.

## Graphical abstract

IBP35, a combination of *Lactiplantibacillus plantarum* IDCC 3501 and *Ligilactobacillus salivarius* IDCC 3551, enhanced macrophage activation in RAW 264.7 cells and improved multiple immune readouts in cyclophosphamide‐induced immunosuppressed mice, including splenocyte proliferation, NK cell activity, cytokine production, macrophage phagocytic activity, and splenic histological features.

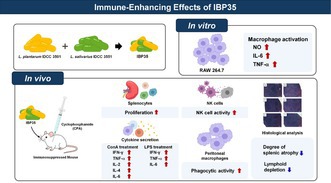

## 1 Introduction

Immune competence is essential for host defense against infections, malignancies, and environmental stressors. However, immune function can be disrupted by a wide range of factors, including aging, chronic stress, infection, nutritional deficiency, and chemotherapeutic agents such as cyclophosphamide (CPA) (Edelman and Zolla‐Pazner 1989; Vivier and Malissen 2005; Zheng et al. 2020; Munteanu and Schwartz 2022; Zhu et al. 2024; Warrick et al. 2025). These immunosuppressive states are associated with higher morbidity and delayed recovery (Khansari et al. 1990; Goodwin 1995; Guggilla et al. 2025), prompting growing interest in nutritional strategies that can support immune function (Wu et al. 2019).

Among dietary interventions, probiotics have been increasingly studied for their immunomodulatory properties. Specific strains have been shown to stimulate macrophage activity, promote natural killer (NK) cell cytotoxicity, and modulate cytokine production, ultimately influencing both innate and adaptive immune responses (Galdeano et al. 2007; Azad et al. 2018; Guo and Lv 2023; Noh et al. 2022). These effects are primarily mediated through interaction with pattern recognition receptors, such as Toll‐like receptors, leading to activation of downstream immune signaling cascades (Chen et al. 2024).

However, several critical limitations persist in the current literature. First, probiotic effects are highly strain specific, and even closely related strains can exhibit divergent immunological profiles (Drago et al. 2010; Van Hemert et al. 2010; Ahmad Kendong et al. 2021). Despite this, several studies rely on species‐ or genus‐level classification, leading to generalizations that overlook intra‐species diversity. This limitation is particularly important because even within a single species, strains can differ markedly in their cytokine‐inducing capacity, underscoring the need for strain‐level evaluation in immunomodulatory screening (Drago et al. 2010). Second, although multi‐strain probiotic formulations are widely used commercially, few studies have rigorously evaluated mixture effects or inter‐strain compatibility (McFarland et al. 2018). Indeed, differences in efficacy between single‐strain and multi‐strain formulations may arise from complex inter‐strain interactions. Therefore, mixed formulations should be experimentally evaluated rather than presumed to provide improved efficacy simply by combining individually effective strains (Ouwehand et al. 2018; McFarland 2021).

In response to these challenges, the development of rationally composed probiotic combinations supported by functional evidence is urgently needed. In this study, we developed IBP35, a 1:1 formulation of *Lactiplantibacillus plantarum* IDCC 3501 and *Ligilactobacillus salivarius* IDCC 3551, selected for their individual immunostimulatory properties. Preliminary findings revealed that the combined formulation induced higher macrophage activation readouts, including nitric oxide (NO) and pro‐inflammatory cytokine production than either strain alone under the tested conditions.

We first assessed the immune‐activating potential of IBP35 in RAW 264.7 macrophages to evaluate its immunomodulatory activity. The formulation was subsequently tested in a CPA‐induced immunosuppressed mouse model to investigate whether it could enhance immune responses under compromised conditions. Parameters including NK cell activity, T cell cytokine production, macrophage enzyme activity, and splenic histopathology were evaluated as functional immune indicators.

This study provides integrated cellular and in vivo evidence supporting the immunomodulatory activity of IBP35. These findings contribute to the scientific foundation for evidence‐based probiotic design and support further investigation of approaches to immune modulation.

## 2 Materials and Methods

### 2.1 Bacterial Strains and Preparation

*L. plantarum* IDCC 3501 and *L. salivarius* IDCC 3551 were obtained from the culture collection of Ildong Bioscience Co. Ltd. (Republic of Korea). Each strain was separately inoculated into a glucose and yeast extract‐based medium and incubated at 37°C for 16 h to achieve optimal growth. Following incubation, bacterial cells were harvested through centrifugation at 3000 × *g* for 10 min at 4°C, washed twice with sterile phosphate‐buffered saline (PBS, pH 7.4), and heat‐killed by autoclaving at 121°C for 15 min. Complete inactivation was verified by plating aliquots on MRS agar and incubating at 37°C for 72 h, with no detectable colony formation. To standardize dosing, each heat‐inactivated preparation was serially diluted in PBS, and the cell number was determined using a Neubauer counting chamber (Marienfeld Superior, Germany). Based on the counted cell number, each strain preparation was adjusted with PBS to a final concentration of 1 × 10^8^ cells/mL. For preparation of IBP35, the heat‐inactivated suspensions of *L. plantarum* IDCC 3501 and *L. salivarius* IDCC 3551 were mixed at a 1:1 (v/v) ratio. Reference strains and isolates were also included for comparative analysis, as detailed in Table 1. Heat‐killed bacterial preparations were used for the in vitro experiments, whereas viable IBP35 preparations were used for the animal study.

**TABLE 1:** List of bacterial strains used in this study.

Species	Strain code	Origin
*Lacticaseibacillus rhamnosus*	GG	Commercial
*Lactiplantibacillus plantarum*	IDCC 3501	Kimchi
	KCTC3108	KCTC culture collection
	KK‐EEL‐009	Sauerkraut
	KK‐EEL‐074	Sauerkraut
*Ligilactobacillus salivarius*	IDCC 3551	Oral cavity of healthy Korean children
	KCTC43133	KCTC culture collection
	KK‐EEL‐347	Human oral cavity

### 2.2 Cell Culture and NO Assay

Murine macrophage RAW 264.7 cells (Korean Cell Line Bank, Seoul, Republic of Korea) were cultured in Dulbecco's Modified Eagle Medium (DMEM; Gibco, Thermo Fisher Scientific, USA) supplemented with 10% heat‐inactivated fetal bovine serum (FBS; Gibco) and 1% penicillin–streptomycin (Gibco) and maintained at 37°C in a humidified incubator with 5% CO₂. Cells were subcultured at 70%–80% confluency and used within a consistent passage range for experiments. For the NO assay, cells were seeded in 96‐well plates at a density of 1 × 10^5^ cells/well and incubated for 24 h. Subsequently, RAW 264.7 cells were treated with bacterial suspension at a final concentration of 1 × 10^7^ cells/mL for 24 h, whereas PBS was used as the control. Culture supernatants were collected, and nitrite concentrations were quantified using the Griess reagent (Sigma‐Aldrich, USA). Briefly, supernatants were mixed with an equal volume of Griess reagent and incubated at room temperature for 10 min protected from light. Absorbance was measured at 540 nm, and NO levels were calculated in μM using a sodium nitrite standard curve.

### 2.3 Enzyme‐Linked Immunosorbent Assay (ELISA)

For cytokine quantification, RAW 264.7 cells were seeded in 96‐well plates at 1 × 10^5^ cells/well and incubated for 18 h. IBP35 was added to RAW 264.7 cells to yield final concentrations of 1 × 10^4^, 1 × 10^5^, 1 × 10^6^, and 1 × 10^7^ cells/mL and incubated for 24 h. Culture supernatants were then collected for cytokine analysis. TNF‐α and IL‐6 levels were measured using commercially available ELISA kits (R&D Systems and Sigma‐Aldrich, USA, respectively) according to the manufacturers' protocols. Absorbance was read using a microplate reader, and cytokine concentrations were calculated from standard curves provided with each kit. Cytokine concentrations are expressed as pg/mL.

### 2.4 Animal Study Design and Treatment Protocol

**FIGURE 1:**
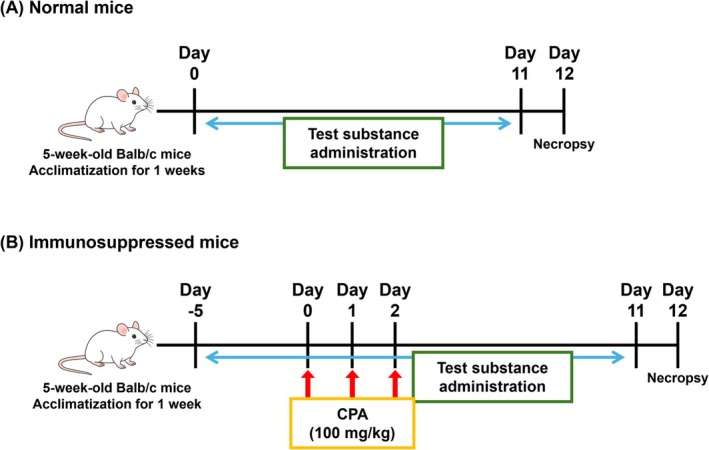
Experimental timeline for IBP35 administration and CPA‐induced immunosuppression IBP35 was administered orally once daily for 12 days, consecutively. In the CPA + IBP35 groups, IBP35 pretreatment began 5 days before the first CPA injection and continued throughout the study. CPA was administered intraperitoneally on Days 0–2. All animals were sacrificed on Day 12 for endpoint analyses.

Six‐week‐old male BALB/c mice (20–23 g) were obtained from the Laboratory Animal Research Center of Chungbuk National University (Cheongju, Republic of Korea) and housed under standard conditions (21°C ± 2°C, 50% ± 20% relative humidity, and a 12 h light/dark cycle) with ad libitum access to food and water. All animal experiments were approved by the Institutional Animal Care and Use Committee of Chungbuk National University (Approval No. CBNUA‐961‐16‐01). After a 1‐week acclimatization period, the mice were randomly assigned to six groups (*n* = 7 per group): normal control (NC), CPA‐only, CPA + IBP35 (1 × 10^5^ CFU/day), CPA + IBP35 (1 × 10^7^ CFU/day), IBP35‐only (1 × 10^5^ CFU/day), and IBP35‐only (1 × 10^7^ CFU/day) to assess potential dose‐dependent effects of IBP35. Except for the NC group, all groups were intraperitoneally administered CPA (Sigma‐Aldrich) at 100 mg/kg body weight once daily for 3 day, consecutively (Days 0–2). IBP35 was administered once daily orally by oral gavage for 12 days, consecutively, at a dosing volume of 10 mL/kg. In the CPA + IBP35 groups, IBP35 administration commenced 5 days prior to the first CPA injection and continued throughout the study (Figure 1 presents the study timeline).

### 2.5 Body Weight and Immune Organ Indices

Mouse body weight was recorded at baseline (Day 0) and at study completion (Day 12). At sacrifice, spleens and thymuses were harvested, blotted dry, and weighed. The immune organ indices were calculated using the following formula:

$$\text{Organ indices}\;\left(\%\right)=\left(\text{Organ weight}\;\left(\mathrm{mg}\right)/\text{Body weight}\;\left(g\right)\right)$$

### 2.6 Splenocyte Isolation and Proliferation Assay

Spleens were aseptically excised and mechanically dissociated through a 40 μm nylon mesh in RPMI‐1640 medium supplemented with 10% FBS, 100 U/mL penicillin, and 100 μg/mL streptomycin. Red blood cells were lysed using a commercial lysis buffer (Hybri‐Max; Sigma‐Aldrich), and the remaining splenocytes were washed and resuspended in complete RPMI medium. Splenocytes were seeded in 96‐well plates (2 × 10^5^ cells/well) and stimulated with lipopolysaccharide (LPS; 20 μg/mL) or concanavalin A (ConA; 5 μg/mL) for 48 h at 37°C. Cell proliferation was assessed using a Cell Counting Kit‐8 (CCK‐8; Dojindo Molecular Technologies, Japan), with absorbance measured at 450 nm using a microplate reader (PowerWave XS; BioTek, USA).

### 2.7 Cytokine Analysis in Splenocyte Supernatants

Splenocytes were seeded in 24‐well plates (5 × 10^6^ cells/well) and stimulated with LPS (20 μg/mL) or ConA (5 μg/mL) for 48 h. Culture supernatants were then collected and analyzed for IL‐2, IL‐4, IL‐6, IFN‐γ, and TNF‐α levels using ELISA kits (Komabiotech, Seoul, Republic of Korea), following the manufacturer's instructions.

### 2.8 Splenic NK Cell Activity Assay

Splenic effector cells (5 × 10^5^ cells/well) were co‐cultured with YAC‐1 target cells at an effector‐to‐target ratio of 50:1 for 4 h. NK cell‐mediated cytotoxicity was evaluated using the CCK‐8 assay. NK cell activity (%) was calculated using the following formula:

$$\mathrm{NK}\;\text{cell activity}\;\left(\%\right)=\left({\mathrm{OD}}_{\text{target}}-\left({\mathrm{OD}}_{\text{target}+\text{effector}}-{\mathrm{OD}}_{\text{effector}}\right)\right)/{\mathrm{OD}}_{\text{target}}\times 100$$

### 2.9 Macrophage Phagocytic Activity Assay

Peritoneal macrophages were elicited through intraperitoneal injection of 3% thioglycollate (1 mL/mouse). After 72 h, peritoneal exudate cells were collected by lavage with cold RPMI‐1640 medium containing 10% FBS. The cells were seeded in 96‐well plates and stimulated with LPS (20 μg/mL) for 24 h at 37°C. Following incubation, the cells were lysed with 0.1% Triton X‐100, and lysosomal enzyme activity was assessed using *p*‐nitrophenyl phosphate as a substrate. Absorbance was read at 405 nm. Macrophage phagocytic activity (%) was calculated using the following formula:

$$\text{Macrophage activity}\;\left(\%\right)=\left({\mathrm{OD}}_{\text{sample}}/{\mathrm{OD}}_{\text{control}}\right)\times 100$$

### 2.10 Histopathological Analysis

Spleen tissues were fixed in 10% neutral‐buffered formalin for 24 h, processed using a tissue processor (VIP‐5 Jr.; Sakura Finetek, Japan), embedded in paraffin, and sectioned at a thickness of 4 μm using a rotary microtome (RM2245; Leica Microsystems, Germany). Sections were stained with hematoxylin and eosin (H&E), and histological features were examined under a light microscope (BX51; Olympus Optical Co., Japan). Key observations included lymphoid depletion, white pulp atrophy, and trabecular architecture.

### 2.11 Statistical Analysis

All experimental data are presented as the mean ± standard deviation (SD). For in vitro assays, experiments were performed in independent replicates and measured in technical replicates per condition. For in vivo experiments, the group size (animals/group) is provided in the figure legends. Homogeneity of variance was assessed using Levene's test. Statistical significance was determined using one‐way analysis of variance (ANOVA), followed by Dunnett's post hoc test. For comparisons in the CPA model, the CPA‐only group served as the reference control, whereas for analyses in normal mice, the NC group served as the reference. Statistical significance was set at *p* < 0.05. All statistical analyses were performed using GraphPad Prism 10 (GraphPad Software, USA).

## 3 Results

### 3.1 Strain‐Specific Immunoactivation and Immunomodulatory Activity of IBP35 in RAW 264.7 Macrophages

**FIGURE 2:**
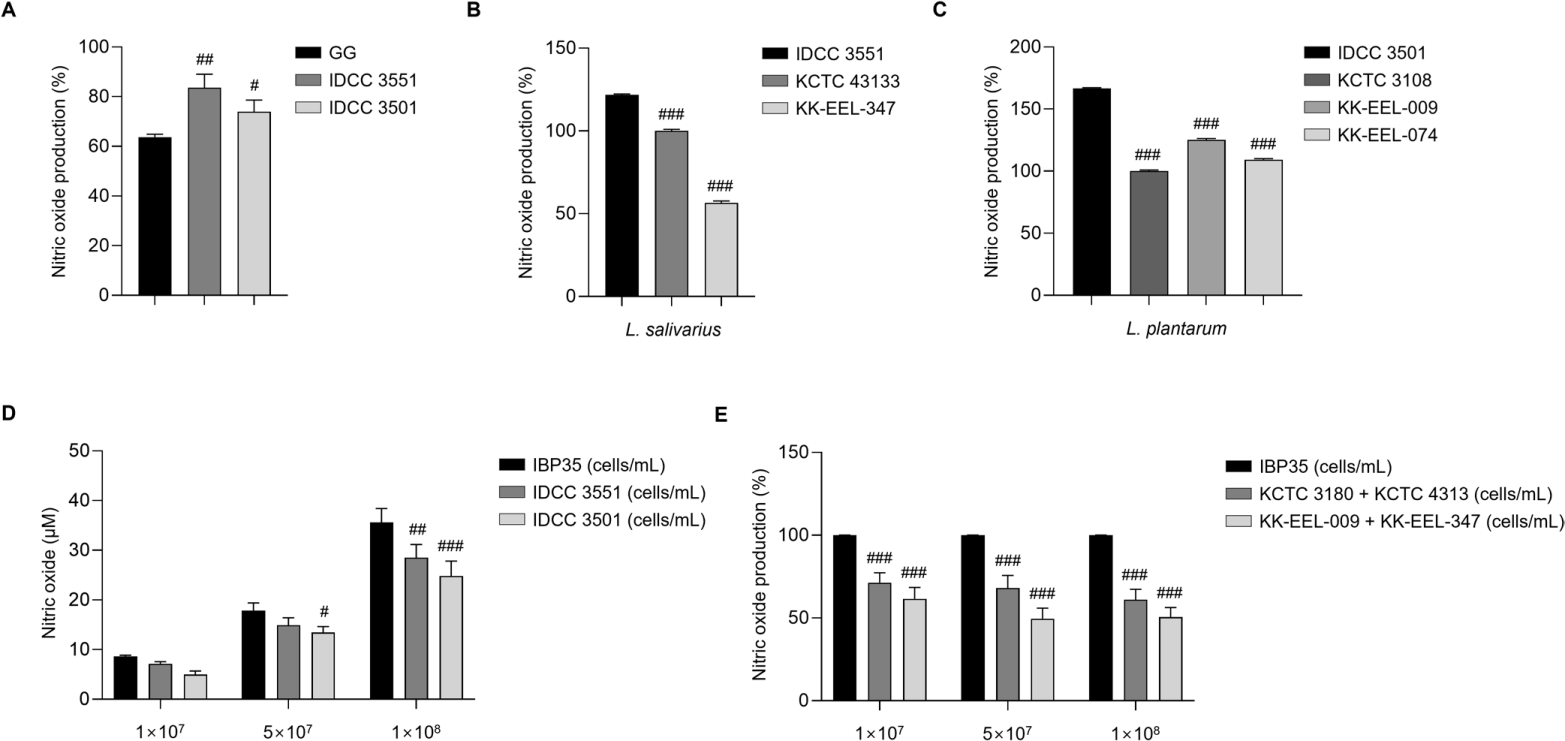
Strain‐specific immune activation and enhanced activity of the combined formulation in RAW 264.7 macrophages. (A) Heat‐killed *L. plantarum* IDCC 3501 and *L. salivarius* IDCC 3551 significantly increased NO production compared with the commercial strain *L. rhamnosus* GG. (B, C) IDCC 3501 and IDCC 3551 exhibited the highest NO‐inducing activity among their respective species, indicating strain‐specific immune potency. (D, E) The combined formulation (IBP35) further enhanced NO production across all tested concentrations, indicating enhanced activity relative to the individual strains under the tested conditions. Data are presented as the mean ± SD (three independent experiments). Statistical analysis was performed using one‐way ANOVA with Dunnett's post hoc test. Statistical significance was set at *p* < 0.05. #*p* < 0.05, ##*p* < 0.01, ###*p* < 0.001.

To assess the immunostimulatory potential of the selected strains, RAW 264.7 macrophages were stimulated with heat‐killed preparations of *L. plantarum* IDCC 3501, *L. salivarius* IDCC 3551, and a commercial probiotic strain, LGG. Both IDCC 3501 and IDCC 3551 induced significantly higher NO production compared with LGG (*p* < 0.01 and *p* < 0.05, respectively), indicating higher immunomodulatory activity in this assay (Figure 2A). Further intra‐species comparison with other *L. plantarum* and *L. salivarius* strains showed that IDCC 3501 and IDCC 3551 elicited the most robust NO response among their respective cohorts (*p* < 0.001), establishing their strain‐specific potency (Figure 2B,C).

We then evaluated NO induction by IBP35, a 1:1 mixture of IDCC 3501 and IDCC 3551. Across all tested concentrations, IBP35 induced higher NO production than either individual strain. At 1 × 10^8^ cells/mL, IBP35 increased NO output by approximately 25% and 43% over IDCC 3551 and IDCC 3501, respectively (*p* < 0.01 and *p* < 0.001; Figure 2D,E). In addition, IBP35 markedly increased TNF‐α and IL‐6 secretion in a concentration‐dependent manner (Figure S1A,B), indicating robust macrophage activation under the tested conditions.

### 3.2 Effects of IBP35 on Body Weight and Immune Organ Indices

To evaluate whether IBP35 influences CPA‐associated changes in lymphoid organs, body weight and lymphoid organ indices (spleen and thymus) were assessed. CPA administration did not significantly affect body weight compared with the normal control (NC) group but markedly reduced the spleen (NC: 3.47 ± 0.32 vs. CPA: 2.78 ± 0.80 mg/g) and thymus (NC: 1.84 ± 0.36 vs. CPA: 1.15 ± 0.24 mg/g) indices (all ###*p* < 0.001 vs. NC). IBP35 at low and high doses did not significantly restore these parameters compared with the CPA group, although a modest recovery trend was observed for the spleen index. In healthy mice, IBP35 did not induce body weight loss or lymphoid organ toxicity (Table 2).

**TABLE 2:** Effects of IBP35 on body weight and immune organ indices.

Group	Body weight (g) on day 12	Spleen index (mg/g)	Thymus index (mg/g)
NC	22.68 ± 1.24	3.47 ± 0.32	1.84 ± 0.36
CPA	22.00 ± 1.14	2.78 ± 0.80^###^	1.15 ± 0.24^###^
CPA + IBP35 (1 × 10^5^ CFU/day)	21.71 ± 0.89^#^	3.11 ± 1.59^###^	1.17 ± 0.28^###^
CPA + IBP35 (1 × 10^7^ CFU/day)	22.42 ± 1.31	3.09 ± 0.43^###^	1.10 ± 0.24^###^
IBP35 (1 × 10^5^ CFU/day)	22.80 ± 0.81	3.45 ± 0.28	1.76 ± 0.30
IBP35 (1 × 10^7^ CFU/day)	23.21 ± 1.11	3.46 ± 0.30	1.80 ± 0.36

*Note:* Data are expressed as the mean ± SD. Body weight (*n* = 21), spleen and thymus indices (*n* = 14). #*p* < 0.05 and ###*p* < 0.001 compared with the NC group.

### 3.3 Effects of IBP35 on Splenocyte Proliferation

**FIGURE 3:**
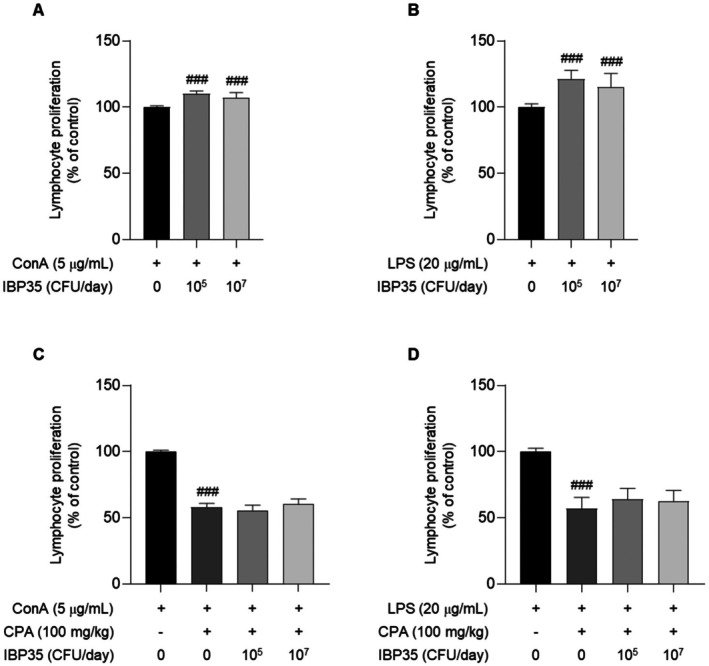
Effects of IBP35 on splenocyte proliferation in normal and immunosuppressed mice. (A, B) In normal mice, splenocyte proliferation in response to ConA (T cell mitogen) and LPS (B cell mitogen) was significantly enhanced after IBP35 treatment at both low and high doses compared with the NC group. (C, D) In CPA‐treated mice, splenocyte proliferation was markedly reduced, confirming successful induction of immunosuppression. IBP35 treatment showed a modest recovery trend, although the changes were not statistically significant. Data are presented as the mean ± SD (seven animals/group). Statistical analysis was performed using one‐way ANOVA with Dunnett's post hoc test. Statistical significance was set at *p* < 0.05. ###*p* < 0.001.

The effects of IBP35 on splenocyte proliferation were evaluated under ConA and LPS stimulation. In normal mice, splenocytes exposed to ConA and LPS exhibited a robust proliferative response, which was further enhanced by IBP35 at both low and high doses (*p* < 0.001 vs. NC; Figure 3A,B). This indicates that IBP35 potentiates both T and B cell responsiveness under physiological conditions.

In CPA‐treated mice, splenocyte proliferation was markedly suppressed, confirming successful immunosuppression. Although IBP35 treatment resulted in modest proliferative recovery, its effects did not reach statistical significance (Figure 3C,D).

### 3.4 Effects of IBP35 on Innate Immune Function in Normal and CPA‐Treated Mice

#### 3.4.1 NK Cell Activity

**FIGURE 4:**
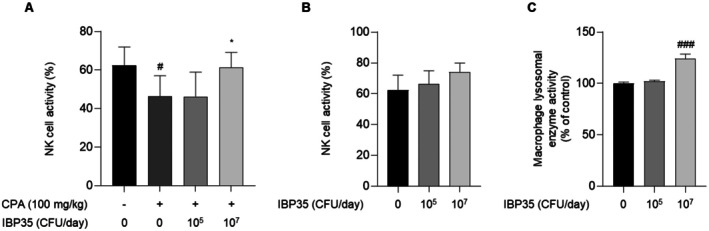
Effect of IBP35 on innate immune function in normal and CPA‐treated mice. (A) CPA treatment significantly reduced splenic NK cell activity compared with the normal control (NC), whereas high‐dose IBP35 significantly increased activity compared with the CPA group, reaching a level comparable to the normal control group. (B) In normal mice, IBP35 increased NK cell activity in a dose‐dependent manner, although the changes were not statistically significant. (C) IBP35 also enhanced lysosomal enzyme activity in peritoneal macrophages following LPS stimulation, with a significant increase observed in the high‐dose group, indicating improved phagocytic function. Data are presented as the mean ± SD (seven animals/group). Statistical analysis was performed using one‐way ANOVA with Dunnett's post hoc test. Statistical significance was set at *p* < 0.05. #*p* < 0.05, ###*p* < 0.001 vs. NC; *p < 0.05 vs. CPA.

CPA administration significantly reduced NK cell activity in splenocytes compared with the NC group (NC: 62.48% ± 9.53% vs. CPA: 46.43% ± 10.64%, *p* < 0.05; Figure 4A). High‐dose IBP35 significantly increased NK cell activity compared with the CPA group (61.31% ± 7.93%, *p* < 0.05 vs. CPA), approaching the level observed in NC. In normal mice, IBP35 treatment increased NK cell activity in a dose‐dependent manner; however, these changes did not reach statistical significance (Figure 4B).

#### 3.4.2 Macrophage Lysosomal Enzyme Activity

To evaluate phagocytic readiness, we measured lysosomal enzyme activity in LPS‐stimulated peritoneal macrophages. IBP35 treatment increased enzyme activity in a dose‐dependent fashion, with the high‐dose group showing a significant enhancement (124.32% ± 4.31%, *p* < 0.001 vs. NC; Figure 4C). These results indicate increased macrophage activity in this assay under the tested conditions.

### 3.5 Cytokine Profiles in Normal and CPA‐Treated Mice

#### 3.5.1 Cytokine Modulation in Normal Mice

**FIGURE 5:**
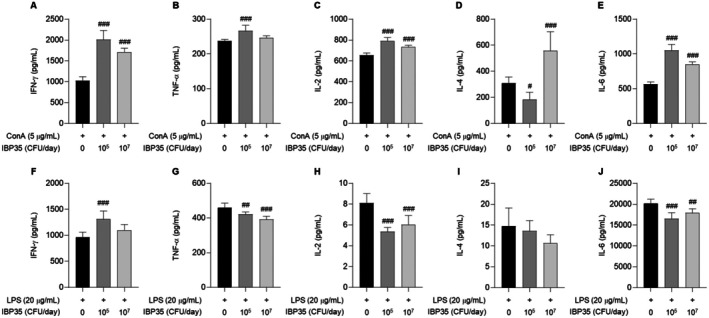
Cytokine modulation by IBP35 in splenocytes from normal mice under ConA or LPS stimulation. (A–E) Under ConA stimulation, IBP35 significantly increased the levels of Th1‐type cytokines IFN‐γ, IL‐2, and TNF‐α, as well as IL‐6, whereas IL‐4 exhibited a biphasic pattern. (F**–**J) Under LPS stimulation, IBP35 mildly increased IFN‐γ levels, while reducing TNF‐α, IL‐2 and IL‐6 levels, with no effect on IL‐4 levels. Data are presented as the mean ± SD (seven animals/group). Statistical analysis was performed using one‐way ANOVA with Dunnett's post hoc test. Statistical significance was set at *p* < 0.05. #*p* < 0.05, ##*p* < 0.01, ###*p* < 0.001.

Under ConA stimulation, IBP35 treatment significantly upregulated Th1‐type cytokines (IFN‐γ, IL‐2, and TNF‐α) in normal mice (*p* < 0.001), indicating a shift toward pro‐inflammatory T cell responses (Figure 5A–C). IL‐6 was also upregulated, whereas IL‐4 exhibited a biphasic pattern—decrease at the low dose and increase at the high dose (Figure 5D,E). In contrast, under LPS stimulation, IBP35 produced a different pattern, characterized by a modest increase in IFN‐γ levels, a decrease in TNF‐α, IL‐2, and IL‐6 levels, and no significant change in IL‐4 levels (Figure 5F–J).

#### 3.5.2 Cytokine Response in CPA‐Treated Mice

**FIGURE 6:**
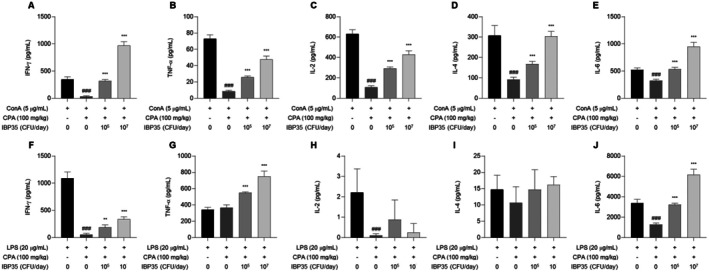
Effect of IBP35 on cytokine production in CPA‐induced immunosuppressed mice. (A**–**E) Under ConA stimulation, CPA significantly suppressed the production of IFN‐γ, IL‐2, TNF‐α, IL‐6, and IL‐4. IBP35 treatment, particularly at the high dose, significantly increased several cytokine levels relative to the CPA group. (F–J) Under LPS stimulation, IBP35 increased IFN‐γ and IL‐6 levels, while effects on IL‐2 and IL‐4 were limited. Data are presented as the mean ± SD (seven animals/group). Statistical analysis was performed using one‐way ANOVA with Dunnett's post hoc test. Statistical significance was set at *p* < 0.05. #*p* < 0.05, ##*p* < 0.01, ###*p* < 0.001.

CPA treatment markedly suppressed ConA‐induced cytokine production. However, IBP35, particularly at the high dose, significantly increased the production of IFN‐γ, IL‐2, TNF‐α, IL‐6, and IL‐4 compared with the CPA‐only group (Figure 6A–E). IFN‐γ and IL‐6 levels significantly increased in a dose‐dependent manner, and TNF‐α levels were elevated compared with the CPA‐only group. In contrast, IL‐2 levels remained significantly lower than normal and exhibited only partial recovery, and IL‐4 showed minimal change (Figure 6F–J). Under LPS stimulation, IBP35 increased IFN‐γ and IL‐6 levels, whereas effects on IL‐2 and IL‐4 were limited.

### 3.6 Histopathological Assessment of Spleen

**FIGURE 7:**
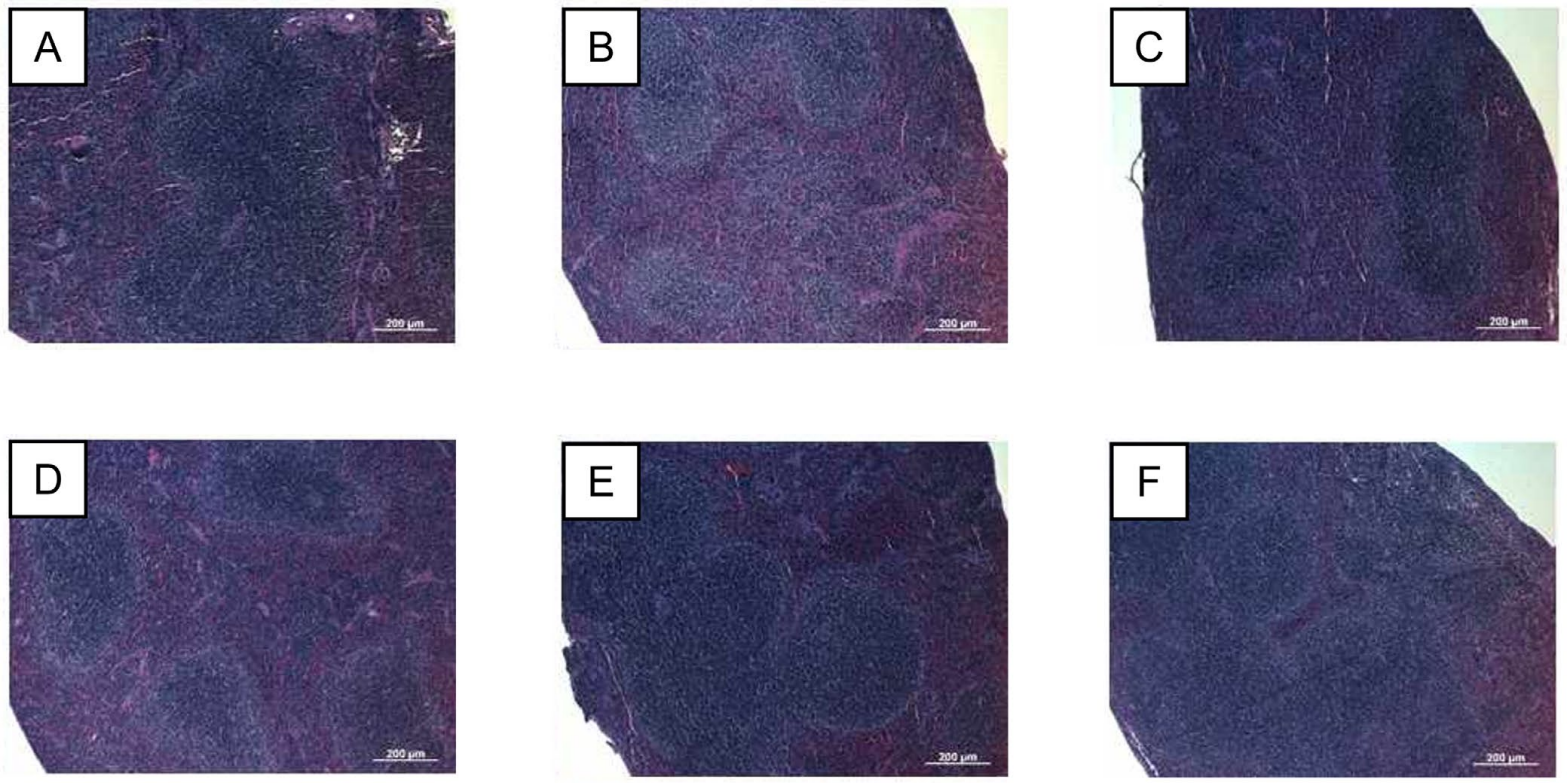
Representative histopathological images of the spleen in normal and immunosuppressed mice. Representative H&E‐stained sections from the (A) NC, (B) CPA, (C) CPA + IBP35 low‐dose, (D) CPA + IBP35 high‐dose, (E) IBP35 low‐dose, and (F) IBP35 high‐dose groups (magnification, ×100).

H&E staining showed that CPA induced splenic atrophy, accompanied by reduced white pulp and lymphoid depletion (Figure 7B). In contrast, IBP35‐treated groups showed preservation of white pulp regions and attenuation of structural alterations compared with the CPA‐only group (Figure 7C,D). Spleens from normal mice treated with IBP35 showed no histopathological abnormalities, supporting the safety of IBP35 administration (Figure 7E,F).

## 4 Discussion

Probiotics and related microbial preparations have been widely investigated as dietary approaches that may help maintain immune homeostasis and modulate host immune responses (Kaur and Ali 2022; Liu et al. 2022; Abbaszadeh et al. 2024). A growing body of evidence indicates that selected strains can influence innate and adaptive immune readouts, including macrophage activation, cytokine production, and natural killer (NK) cell activity (Galdeano et al. 2007; Azad et al. 2018; Guo and Lv 2023; Noh et al. 2022). However, probiotic effects are strongly strain dependent, and even closely related strains can elicit divergent immune profiles (Drago et al. 2010; Kim 2018; Yoo et al. 2020; McFarland et al. 2018). Consistent with this concept, our strain‐level comparison showed that *Lactiplantibacillus plantarum* IDCC 3501 and *Ligilactobacillus salivarius* IDCC 3551 exhibited stronger immunostimulatory activity than other strains within the same species, thus supporting the higher functional potency of these IDCC strains under the tested conditions.

Although the physiological benefits of single probiotic strains are well recognized, the efficacy of strain combinations remains incompletely defined. Combining different probiotic strains is widely used to improve beneficial activities; however, formulation‐level outcomes are not always predictable from species‐ or strain‐level information alone and therefore require empirical evaluation with transparent reporting of endpoint‐specific effects (McFarland et al. 2018). Previous studies have largely focused on single strains, and comparisons across strains at the species and formulation levels have been limited. In this study, we developed IBP35, a defined 1:1 formulation of *L. plantarum* IDCC 3501 and *L. salivarius* IDCC 3551 and evaluated its immunomodulatory activity using linked in vitro and in vivo readouts. We compared multiple strains within each species and then assessed the defined formulation to characterize endpoint‐specific effects under the tested conditions.

Macrophage NO production and cytokine secretion are commonly used in vitro indicators of innate immune activation. In this study, IBP35 increased NO, TNF‐α, and IL‐6 production in a dose‐dependent manner and showed higher macrophage activation readouts than either constituent strain alone, indicating increased activity of the IBP35 formulation relative to single‐strain treatments under the tested conditions. Consistent with prior formulation‐level observations, mixture effects are not always readily inferred from individual components. For example, a three‐strain probiotic mixture (ID‐JPL934) suppressed LPS‐induced NO and pro‐inflammatory cytokine outputs more effectively than each member strain tested individually in RAW 264.7 macrophages (Choi and Moon 2023). Similarly, a mixed *Bacillus subtilis* – *Lactobacillus sakei* fermentation product induced stronger NO production than single‐microbe fermentates and increased TNF‐α and IL‐6 secretion in RAW 264.7 macrophages (Yoon et al. 2025). Taken together, these findings support that IBP35 elicits consistent macrophage activation readouts and highlight that measurable immune responses can vary across strains and formulations, consistent with the broader concept of strain specificity in probiotic immunology (McFarland et al. 2018). However, additional studies are required to clarify determinants of formulation‐level activity and to improve reproducibility across preparations and experimental settings.

The cyclophosphamide‐induced immunosuppressed mouse model provides a useful framework to evaluate whether IBP35 modulates immune function under compromised conditions. In our study, CPA reduced NK cell activity and suppressed mitogen‐stimulated cytokine production. Under these conditions, high‐dose IBP35 significantly increased NK cell activity compared with the CPA‐only group, approaching the level observed in normal controls. Histologically, IBP35 reduced lymphoid depletion in CPA‐treated mice. At the same time, our results highlight that immunomodulatory effects are endpoint dependent. Although NK activity and several immune readouts showed clearer changes in CPA‐treated mice, splenocyte proliferation indices showed modest differences. This endpoint‐specific pattern is not unusual in probiotic immunology, where the responsiveness of immune compartments can differ based on stimulus type, immune status, and the measured outcome (Mohamadzadeh et al. 2005; McFarland et al. 2018). Similar patterns have been reported in other CPA‐induced immunosuppression models (Salva et al. 2014; Kim et al. 2023; Ma et al. 2023). For example, a three‐strain complex probiotic increased several immune indices and serum cytokines at medium and high doses, whereas IFN‐γ showed a significant increase only at the highest dose, illustrating that formulation effects can differ across readouts and dosing conditions (Ma et al. 2023). Similarly, in a CPA‐induced model, a mixture of *Limosilactobacillus fermentum* strains showed the highest effects for NK cytotoxicity and splenocyte proliferation, whereas CD4+/CD8+ T‐cell proportions were not significantly changed, further supporting readout‐dependent responses (Kim et al. 2023). Accordingly, these findings reveal that IBP35 may preferentially influence selected functional immune readouts under immunosuppressed conditions, with the magnitude of response varying by endpoint and dose.

Cytokine profiling provided further insights into the immunoregulatory characteristics of IBP35. In normal mice, IBP35 enhanced Th1‐associated cytokine production (IFN‐γ, IL‐2, and TNF‐α) in response to ConA stimulation and concomitantly increased IL‐6 levels. In contrast, under LPS stimulation, IBP35 reduced TNF‐α, IL‐2, and IL‐6 levels while modestly increasing IFN‐γ levels at the lower dose. This bidirectional regulation is consistent with earlier observations that *Lactobacillus* strains can induce highly context‐dependent effects on Th1/Th2 balance and innate cytokine output, sometimes favoring Th1 polarization and in other circumstances dampening overactive inflammatory responses through differential activation of antigen‐presenting cells and T lymphocytes (Mohamadzadeh et al. 2005). In CPA‐treated mice, IBP35 increased ConA‐induced cytokine production relative to CPA‐only controls, indicating improved responsiveness in this stimulation context. Collectively, these findings indicate that IBP35 modulates immune readouts in a manner shaped by both host immune status.

The immune activation observed after IBP35 treatment may be interpreted as a process in which early cytokine responses in macrophages and antigen‐presenting cells (APCs) are linked to downstream adaptive immune outputs. In this study, IBP35 increased NO, TNF‐α, and IL‐6 in RAW 264.7 macrophages, and the expression of these mediators is generally regulated by innate immune gene programs involving canonical pathways such as NF‐κB and MAPK (Tabarsa et al. 2020; Lee et al. 2022). Probiotic bacterial preparations, as exemplified by IBP35, may contain immunogenic cell‐associated molecules, including lipoteichoic acid (LTA), exopolysaccharides (EPS), and peptidoglycan, which can contribute to innate immune sensing through pattern‐recognition receptors (such as TLR2/6 and NOD‐like receptors) that converge on NF‐κB/MAPK‐regulated transcriptional programs (Angelin and Kavitha 2020; Paveljšek et al. 2021; Furnari et al. 2025). For example, Lactobacillus‐derived exopolysaccharides have been shown to activate NF‐κB/MAPK signaling in RAW 264.7 macrophages (Gao et al. 2022), and Lactobacillus‐derived lipoteichoic acid has been reported to modulate TLR–MyD88–MAPK/NF‐κB signaling (Zhang et al. 2023); moreover, several Lactobacillus strains have been shown to induce iNOS/NO and cytokine expression through NF‐κB and MAPK pathways in RAW 264.7 macrophages (Park et al. 2025). TNF‐α can promote the expression of inflammatory cytokines and chemokines and support early immune cell recruitment, while also enhancing dendritic cell maturation, antigen presentation, and co‐stimulatory signaling required for T‐cell activation. IL‐6 serves as a key bridge between innate and adaptive immunity and can influence B‐cell antibody production and T‐cell differentiation environments. Accordingly, these macrophage/APC‐associated cytokines observed after IBP35 treatment may contribute to the ConA‐associated increases in Th1‐type cytokines (IFN‐γ, IL‐2, and TNF‐α) and to changes in NK activity observed in vivo through cytokine‐mediated crosstalk. In contrast, the distinct cytokine pattern under LPS stimulation (reduced TNF‐α/IL‐6 with modest IFN‐γ changes) may reflect stimulus‐dependent regulation of inflammatory cytokine output. However, additional studies with direct mechanistic assays are required to confirm the underlying pathways.

An additional strength of this study is that immune modulation was evaluated in both healthy and immunosuppressed settings. In healthy mice, IBP35 modulated immune readouts without evidence of overt toxicity or excessive inflammatory pathology at the tested doses, as indicated by the absence of body‐weight loss, no apparent lymphoid organ toxicity, and unremarkable splenic histology in IBP35‐only groups. These observations support that measurable changes in immune readouts can occur without gross inflammatory damage under the experimental conditions used.

This study has several limitations. First, the administration period was relatively short (12 days), and longer‐term studies are needed to assess durability and safety. Second, the in vivo evaluation was limited to a single mouse strain/sex and a single immunosuppression paradigm; validation across additional models and immune challenges would strengthen generalizability. Third, mechanistic validation and microbiome/metabolite profiling were not conducted, limiting conclusions about underlying pathways and potential microbiota‐mediated contributions. Finally, because some endpoints showed modest or non‐significant effects in CPA‐treated mice, conclusions should remain endpoint specific and avoid generalization across all immune outcomes. To support clinical translation, it is also important to determine whether the immune readout changes observed in the mouse model translate into clinically meaningful outcomes in humans. Moreover, responses may vary depending on the target population's health status and individual baseline characteristics. Accordingly, further clinical studies are needed with clearly defined, clinically relevant endpoints and with well‐specified target populations and use conditions.

Collectively, the findings of this study provide foundational evidence that IBP35 exhibits immunomodulatory activity across multiple readouts in cellular assays and a CPA‐induced immunosuppressed mouse model, thereby supporting further mechanistic and translational evaluation.

## 5 Conclusion

This study provides evidence that IBP35, a combination of *L. plantarum* IDCC 3501 and *L. salivarius* IDCC 3551, exhibits immunomodulatory activity across multiple immune readouts. In vitro, IBP35 significantly enhanced macrophage activation, NO production, and pro‐inflammatory cytokine release, showing higher macrophage activation readouts than either individual strain under the tested conditions. In vivo, IBP35 improved several immune readouts in CPA‐induced immunosuppressed mice, including cytokine production, NK cell activity, macrophage enzyme activity, and splenic histological features; however, splenocyte proliferation showed only modest, non‐significant improvement.

Collectively, these findings reveal that IBP35 may be a potential candidate for immune support. Its capacity to modulate immune responses under physiological conditions and to improve selected immune readouts under immunosuppressed conditions supports further investigation as a functional ingredient.

Future research should extend these findings through longer‐term evaluations, dose‐ranging studies, and targeted mechanistic investigations. In addition, to support clinical translation, human studies using clearly defined, clinically meaningful endpoints are required, and variability by population health status and baseline immune characteristics should be considered. If validated in translational contexts, IBP35 could contribute to the growing field of immunonutrition and support immune function in both healthy and immunocompromised populations.

## Author Contributions

**Jin Seok Moon:** conceptualization, project administration, writing – review and editing. **Han Bin Lee:** writing – original draft, investigation, formal analysis. **Jinho Lee:** methodology, data curation. **Kyuho Jeong:** resources, supervision. **Young Hoon Jung:** validation, visualization.

## Conflicts of Interest

Han Bin Lee, Jinho Lee, and Jin Seok Moon are affiliated with Ildong Bioscience Co. Ltd. Kyuho Jeong is affiliated with Ildong Pharmaceutical Co. Ltd. All other authors declare no competing interests.

## Supporting information


**Figure S1:** Concentration‐dependent effects of IBP35 on cytokine production in RAW 264.7 macrophages. RAW 264.7 macrophages were treated with IBP35 for 24 h, and cytokine levels in the culture supernatants were quantified using ELISA. (A, B) IBP35 significantly increased TNF‐α (A) and IL‐6 (B) secretion in a dose‐dependent manner. Data are presented as the mean ± SD (*n* = 3). Statistical analysis was performed using one‐way ANOVA with Dunnett's post hoc test. *p* < 0.05 was considered statistically significant. #*p* < 0.05, ##*p* < 0.01, ###*p* < 0.001.

## Data Availability

The data that support the findings of this study are available from the corresponding author upon reasonable request.
