# Supplementary material for: Immune‐Enhancing Effects of IBP35, a Combination of Lactiplantibacillus plantarum and Ligilactobacillus salivarius, in Macrophages and Cyclophosphamide‐Induced Immunosuppressed Mice
Source: Food Sci Nutr. 2026 Apr 29;14(5):e71831. doi: 10.1002/fsn3.71831 (PMC13126242; doi:10.1002/fsn3.71831)
Supplement: Supplementary file 1 — Figure S1: Concentration‐dependent effects of IBP35 on cytokine production in RAW 264.7 macrophages. RAW 264.7 macrophages were treated with IBP35 for 24 h, and cytokine levels in the culture supernatants were quantified using ELISA. (A, B) IBP35 significantly increased TNF‐α (A) and IL‐6 (B) secretion in a dose‐dependent manner. Data are presented as the mean ± SD (n = 3). Statistical analysis was performed using one‐way ANOVA with Dunnett's post hoc test. p < 0.05 was considered statistically significant. #p < 0.05, ##p < 0.01, ###p < 0.001. [file FSN3-14-e71831-s001.docx]

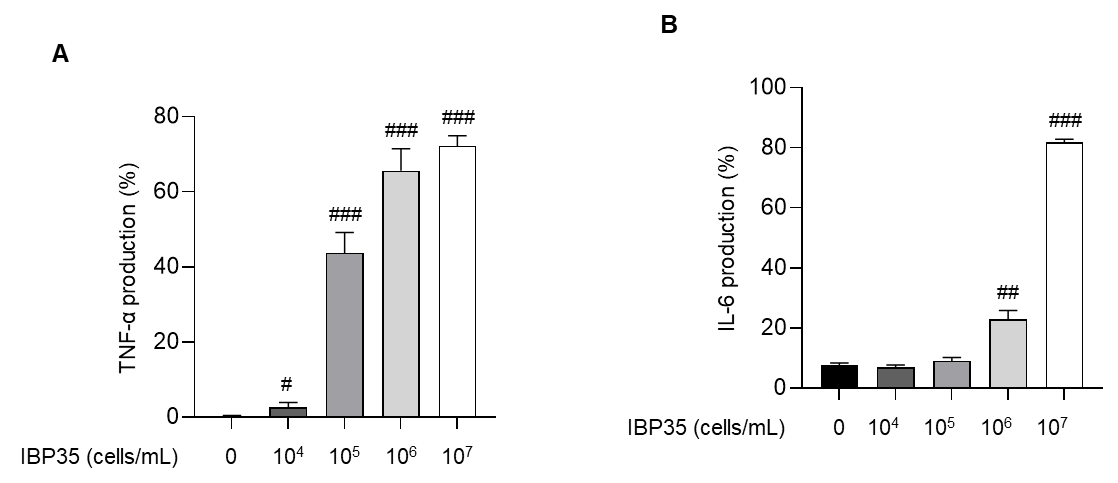


**Supplementary Figure S1. Concentration-dependent effects of IBP35 on cytokine production in RAW 264.7 macrophages.**RAW 264.7 macrophages were treated with IBP35 for 24 h, and cytokine levels in the culture supernatants were quantified using ELISA. (A, B) IBP35 significantly increased TNF-α (A) and IL-6 (B) secretion in a dose-dependent manner. Data are presented as the mean ± SD (*n* = 3). Statistical analysis was performed using one-way ANOVA with Dunnett’s post hoc test. *p* < 0.05 was considered statistically significant. #*p* < 0.05, ##*p* <0.01, ###*p* <0.001.
